# Comparative performance analysis of interventional devices for the treatment of ischemic disease in below-the-knee lesions: a systematic review and meta-analysis

**DOI:** 10.1007/s12928-021-00758-7

**Published:** 2021-02-06

**Authors:** Emi Kearon Matsuoka, Terumitsu Hasebe, Ryota Ishii, Naoki Miyazaki, Kenzo Soejima, Kiyotaka Iwasaki

**Affiliations:** 1 Cooperative Major in Advanced Biomedical Sciences, Graduate School of Advanced Science and Engineering, Joint Graduate School of Tokyo Women’s Medical University, Waseda University, 2-2 Wakamatsu-cho, Shinjuku, Tokyo, 162-8480 Japan; 2 Division of Translational Research, Clinical and Translational Research Center, Keio University Hospital, Tokyo, Japan; 3 Vascular & Interventional Center/Department of Radiology, Tokai University Hachioji Hospital, Tokai University School of Medicine, Tokyo, Japan; 4 Biostatistics Unit, Clinical and Translational Research Center, Keio University Hospital, Tokyo, Japan; 5 Department of Modern Mechanical Engineering, School of Creative Science and Engineering, Waseda University, Tokyo, Japan; 6 Department of Integrative Bioscience and Biomedical Engineering, Graduate School of Advanced Science and Engineering, Waseda University, Tokyo, Japan

**Keywords:** Below-the-knee (BTK), Drug-eluting stent (DES), Drug-coated balloon (DCB), Bare-metal stent (BMS), Meta-analysis

## Abstract

This meta-analysis aimed to evaluate the device performance of conventional balloon catheters (POBA), drug-coated balloons (DCB), bare-metal stents (BMS), and drug-eluting stents (DES) in below-the-knee (BTK) ischemic lesions with regard to lesion characteristics. Online searches of PubMed, Web of Science, and Cochrane databases (2010–2019) were conducted for each of the test devices. Primary patency rates (pp) and major amputation rates 1 year after the use of each device were analyzed using a random-effects meta-analysis model. Meta-regression analysis was conducted to test associations between the outcomes and lesion characteristics. The analysis included 18 studies reporting on 24 separate cohorts comprising 2,438 patients. DES demonstrated the best pp among the test devices (83.6%; 95% confidence interval = 78.4–88.8%, studies = 8; *I*^2^ = 66%, *P* = 0.005). A negative coefficient between lesion length and pp (*P* = 0.002) was obtained. The ratio of critical limb ischemia (CLI) patients impacted the amputation rates (*P* = 0.031), whereas no statistically significant difference was found between the devices. DES showed favorable pp in BTK lesions; however, as the lesion lengths using DES were short, pp in long lesions still needs to be evaluated. Shorter lesions gained better pp. A higher ratio of CLI patients resulted in increased amputation rates.

## Introduction

The efficacy of endovascular therapy (EVT) in below-the-knee (BTK) ischemic lesions has been recognized in recent years [1–3]. However, the quality of clinical outcomes after EVT for BTK lesions is still unsatisfactory compared to that for above-the-knee lesions due to the anatomic challenges and limited device options for BTK lesions [3]. EVT devices commonly used in BTK lesions include plain conventional balloon catheters (POBAs), drug-coated balloons (DCBs), bare-metal stents (BMSs), and drug-eluting stents (DESs). Of these, only POBAs are approved for BTK lesions in the United States and in Japan. All devices are currently available in the European Union (EU); however, the DESs that are used are adapted from coronary or superficial femoral artery stents. The optimal EVT device for the treatment of BTK lesions remains a topic of debate.

Several meta-analyses have demonstrated the advantages of using coronary DES compared to other EVT devices in BTK lesions [1–4]. However, device selection for BTK lesions with regard to lesion characteristics has not been sufficiently investigated.

In this study, a comprehensive literature analysis was conducted to compare the primary patency rates (pp) and major amputation rates as indexes of clinical performance of EVT devices in BTK lesions. Subsequently, the associations between the outcomes and lesion characteristics, including average lesion lengths, existence of chronic total occlusion (CTO), and existence of critical limb ischemia (CLI) were examined.

## Materials and methods

### Search methods

Studies that analyzed the clinical performance of EVT for the treatment of ischemic lesions using POBA, DCB, BMS, or DES were investigated. Database searches of PubMed (Medline), Web of Science, and Cochrane were conducted on November 26, 2019 for articles published between January 1, 2010 and November 26, 2019. The search words included “PAD,” “lower limb,” “endovascular,” “POBA,” “balloon,” “plain balloon,” “conventional balloon,” “PTA,” “DCB,” “drug coated balloon,” “stent,” “BMS,” “drug eluting stent,” and “DES.”

### Trial selection and data extraction

The data selection process for this study complied with the Preferred Reporting Items for Systematic Reviews and Meta-analysis (PRISMA) statement. To extract valid data to compare pp and amputation rates in BTK lesions, the following study selection criteria were established: (i) clinical studies for peripheral artery disease (PAD) which were treated with any of the four test devices were included; (ii) studies for acute ischemia were excluded; (iii) patient number was at least 10; (iv) 1-year pp data were available; (v) for POBA and DCB, provisional stenting with BMS was acceptable for up to a maximum of 10% of the cases; (vi) for BMS and DES, pre- or post-expansion with POBA was acceptable; (vii) studies for in-stent restenosis (ISR) were excluded; (viii) target lesions were BTK, including BTK popliteal artery, anterior tibial artery, peroneal artery, and posterior tibial artery.

Review papers, meta-analyses, and pooled analyses were excluded. Study selection was made by 2 independent reviewers (E.M. and K.I.). After the data selection process, pp and major amputation rates at 1 year and information regarding lesion characteristics were collected from the included articles. The lesion characteristic variables used for quantitative synthesis in this study were as follows: average lesion length, the ratio of CTO lesions, and the ratio of CLI patients (Rutherford class 4–6) [5].

### Risk of bias and applicability assessment of primary studies

The Cochrane Collaboration’s tool for assessing the risk of bias (QUADAS-2) was employed to assess the risk of bias and the applicability of each study [6]. QUADAS-2 consists of four key domains: patient selection; conduct or interpretation of index test; reference standard that defines target conditions; and patient flow and timing of the test. All domains were assessed for risk of bias and the first three were assessed for concerns regarding applicability. The risk of bias and concerns regarding applicability were graded as low, high, or unclear. The assessments were performed by 2 independent reviewers (E.M. and K.I.), with divergences resolved after mutual consensus.

### Statistical analyses

The OpenMetaAnalyst for Sierra (10.12) was utilized to conduct statistical analysis [7]. Categorical variables are summarized as frequencies (percentages). Statistical pooling of the studies was performed according to a random-effect model for computing incidence estimates with 95% confidence intervals (CI). Statistical heterogeneity was tested with *I*^2^; 0% indicates no observed heterogeneity and larger values indicate increasing heterogeneity. To account for differences in lesion characteristics across studies, meta-regression analyses were conducted to test associations between the outcomes and lesion characteristics. Two-sided *P* values < 0.05 were considered as significant.

## Results

### Included data for the quantitative analysis

**Fig. 1:**
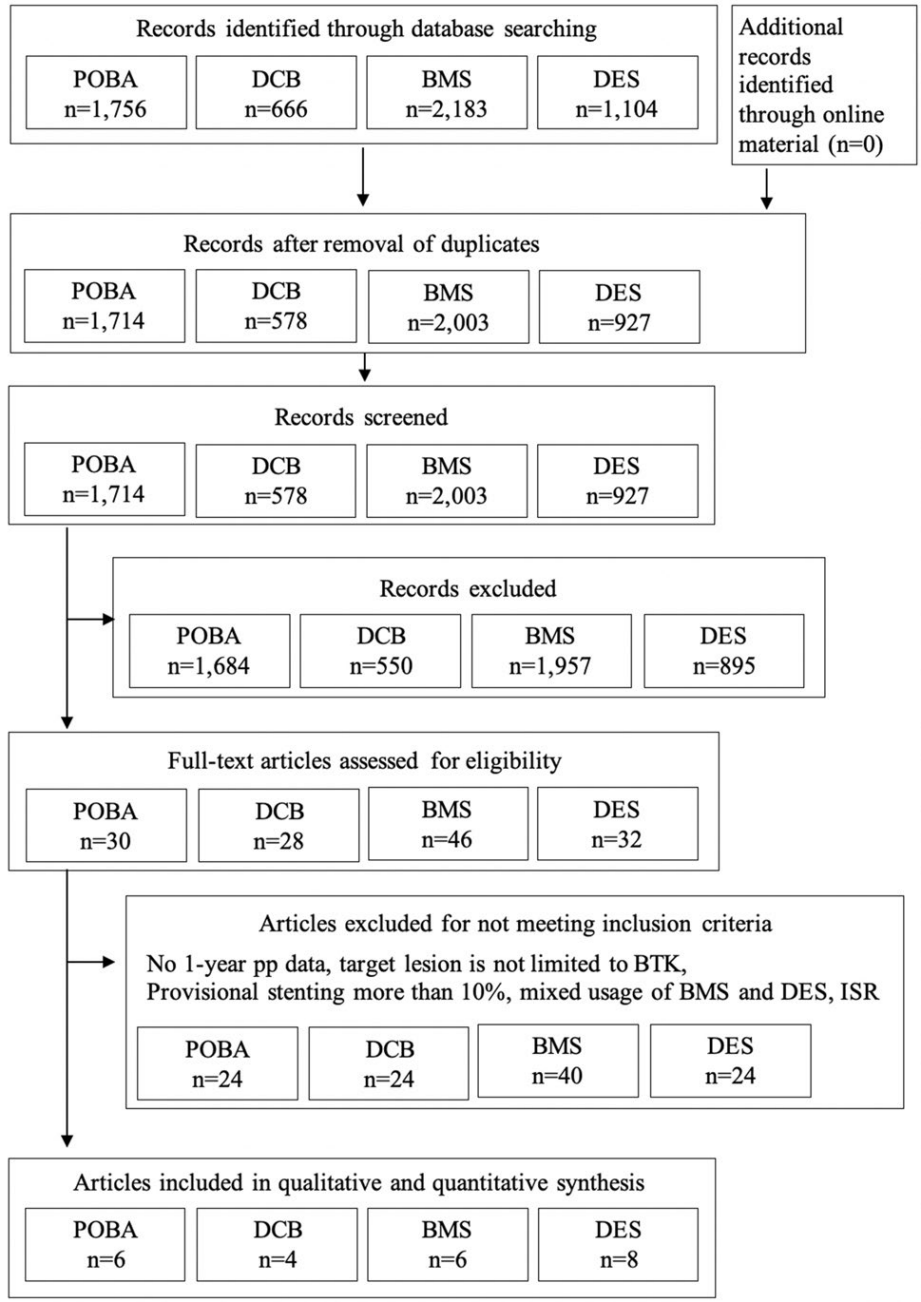
Preferred reporting items for systematic reviews and meta-analysis flowchart. Literature search and study selection process. *POBA* conventional balloon catheters, *DCB* drug-coated balloons, *BMS* bare-metal stents, *DES* drug-eluting stents, *BTK* below-the-knee, *ISR* in-stent restenosis

The total number of articles extracted from the 3 online databases was as follows: 1756 for POBA, 666 for DCB, 2183 for BMS, and 1104 for DES. After the selection process, 18 studies reporting on 24 separate cohorts comprising 2438 patients were included in this analysis (Fig. 1) [8–25]. A list of the cohorts used for this study with the risk of bias and applicability assessment is provided in Table 1. Average lengths of the lesions treated with the stents (BMS, DES; 21.1 mm–52.7 mm) were shorter than those of the lesions treated with the balloons (POBA, DCB; 43.2 mm–131.0 mm). All of the DESs used were coronary stents. All of the BMSs used were coronary or peripheral stents of which the smallest stent diameter was 4.0 mm. In a majority of the studies, pp was defined as freedom from binary restenosis which was determined by the peak systolic velocity ratio (PSVR) detected by duplex ultrasonography. The typical PSVR cut-off value for being patent or restenosed was 2.4 or 2.5 among the included studies [26, 27].

**Table 1:** List of the cohorts used for this study with the risk of bias and applicability assessment

												Risk of bias			Applicability concerns			
device	trial	device name	study type	year of publish	number of patients enrolled	average lesion length/mm	%CTO lesion	%CLI patients	% calcified lesions	DAPT period /months	primary patency /restenosis cut-off value definition	patient selection	index test for pp	reference standard for pp	flow and timing	patient selection	index test for pp	reference standard for pp
POBA	DEBATE-BTK (POBA)	–	RCT	2013	67	131	82%	100%	28%	1mo	DUS PSVR 2.5 or 50% restenosis under angiography	L	L	L	L	L	L	L
POBA	ETAP study (POBA)	–	RCT	2013	127	43.2	33%	21%	-	1mo	DUS PSVR 2.4	L	L	L	L	L	L	L
POBA	Todd KE Jr. et al.	–	retrospective study	2013	339	-	44%	100%	–	–	uninterrupted patency post-procedure by DUS, ABI, angiography	L	U	U	U	L	L	L
POBA	BIOLUX P-II (POBA)	–	RCT	2015	36	115	-	78%	18%	1mo	50% restenosis by QVA without TLR	L	L	L	L	L	L	L
POBA	Haddad SE et al. (POBA)	Oceanus 35	RCT	2017	45	–	–	100%	–	3mo	restenosis defined by luminal diameter > 50% according to the worst angiographic view or by DUS PSVR ≥ 2.5	L	L	L	L	L	L	L
POBA	ANJE registry (POBA)	-	registry	2018	110	53.1	30%	100%	–	3mo (51%)*	50% stenosis	L	L	L	L	L	L	L
DCB	DEBATE-BTK (DCB)	IN.PACT Amphirion	RCT	2013	65	129	78%	100%	25%	1mo	DUS PSVR 2.5 or 50% restenosis under angiography	L	L	L	L	L	L	L
DCB	BIOLUX P-II (DCB)	Passeo-18 lx	RCT	2015	36	113.1	21%	78%	44%	1mo	50% restenosis by QVA without TLR	L	L	L	L	L	L	L
DCB	Haddad SE et al. (DCB)	Luminor 14	RCT	2017	48	–	–	100%	–	3mo	restenosis defined by luminal diameter > 50% according to the worst angiographic view or by DUS PSVR ≥ 2.5	L	L	L	L	L	L	L
DCB	APOLLO Trial	ELUTAX SV	registry	2019	164	71.2	43%	96%	27%	1mo**	sufficient flow by DUS without TLR	L	L	U	L	L	L	L
BMS	YUKON-BTX (BMS)	yukon BMS (BX)	RCT	2012	79	31	22%	42%	–	6mo	DUS PSVR 2.4	L	L	L	L	L	L	L
BMS	Golts JP et al.	SUPERA (4-6 mm diameter)	retrospective study	2012	40	–	–	100%	–	1mo	50% restenosis by DUS or DSA	U	U	L	U	L	L	L
BMS	ETAP study (BMS)	LifeStent (6-8 mm diameter)	RCT	2013	119	41.3	33%	21%	–	1mo	DUS PSVR 2.4	L	L	L	L	L	L	L
BMS	EXPAND	Astron Pulsar, Pulsar-18 stent	RCT	2015	45	≦190	–	–	–	1mo	DUS PSVR 2.4 or angiography	L	L	L	L	L	L	L
BMS	ANJE registry (BMS)	SX, BX	registry	2018	169	39.4	30%	100%	–	3mo (51%)*	50% stenosis	L	L	L	L	L	L	L
BMS	Potoczny PW et al.	Jaguar (SX)	retrospective study	2018	172	–	–	100%	–	–	–	L	U	U	U	L	L	L
DES	Balzer JO et al.	Cypher	registry	2010	114	46	22%	100%	–	6mo	uninterrupted patency with no procedures performed	L	L	U	L	L	L	L
DES	McMillan WD et al.	Taxus	retrospective study	2010	52	24	100%	100%	–	6mo	DUS and ABI	L	U	U	U	L	L	L
DES	Rastan A et al.	Cypher Select	registry	2010	146	36.6	30%	45%	–	6mo	70% restenosis by angiography or DUS PSVR 3.4	L	L	L	L	L	L	L
DES	YUKON-BTX (DES)	YUKON	RCT	2012	82	31	23%	51%	–	6mo	DUS PSVR 2.4	L	L	L	L	L	L	L
DES	Werner M et al.	Cypher Select	retrospective study	2012	158	33.6	26%	44%	–	6mo	50% restenosis by angiography	L	U	L	U	L	L	L
DES	Stabile E et al.	BioMatrix-Biolims A9	retrospective study	2016	30	23.5	–	67%	–	6mo	absence of CD-TLR and binary restenosis by angiography or DUS	U	U	L	U	L	L	L
DES	Etna Registry	Xience Prime	registry	2017	122	52.7	68%	100%	65%	3mo	DUS PSVR 2.4	L	L	L	L	L	L	L
DES	PADI trial	TAXUS Liberté	RCT	2017	73	21.1	3%	100%	–	6mo	DUS PSVR 2.0	L	L	L	L	L	L	L

*POBA* plain conventional balloon catheter, *DCB* drug-coated balloon, BMS: bare-metal stent, *DES* drug-eluting stent, *SX* self-expanding stent, *BX* balloon-expandable stent, *RCT* randomized controlled trial, *CTO* chronic total occlusion, *CLI* critical limb ischemia, *DAPT* dual-anti-platelet therapy, *DUS* duplex ultrasonography, *PSVR* peak systolic velocity ratio, *ABI* ankle-brachial index, *TLR* target lesion revascularization, *CD-TLR* clinically driven target lesion revascularization, *pp* primary patency rates, *L* Low, *U* Unclear

*Rest of the patients had aspirin or clopidogrel

**65% was under DAPT at 6 months

Most of the retrospective studies did not well define the reference standard of primary patency or how follow-up tests were conducted and were marked as “Unclear” in the corresponding domain of bias risk assessment. Index test domain and reference standard domain risk of bias and applicability concerns for major amputation rates were assessed as “Low” as they are generally accepted as amputations at or proximal to the ankle.

### pp in each device group

**Fig. 2:**
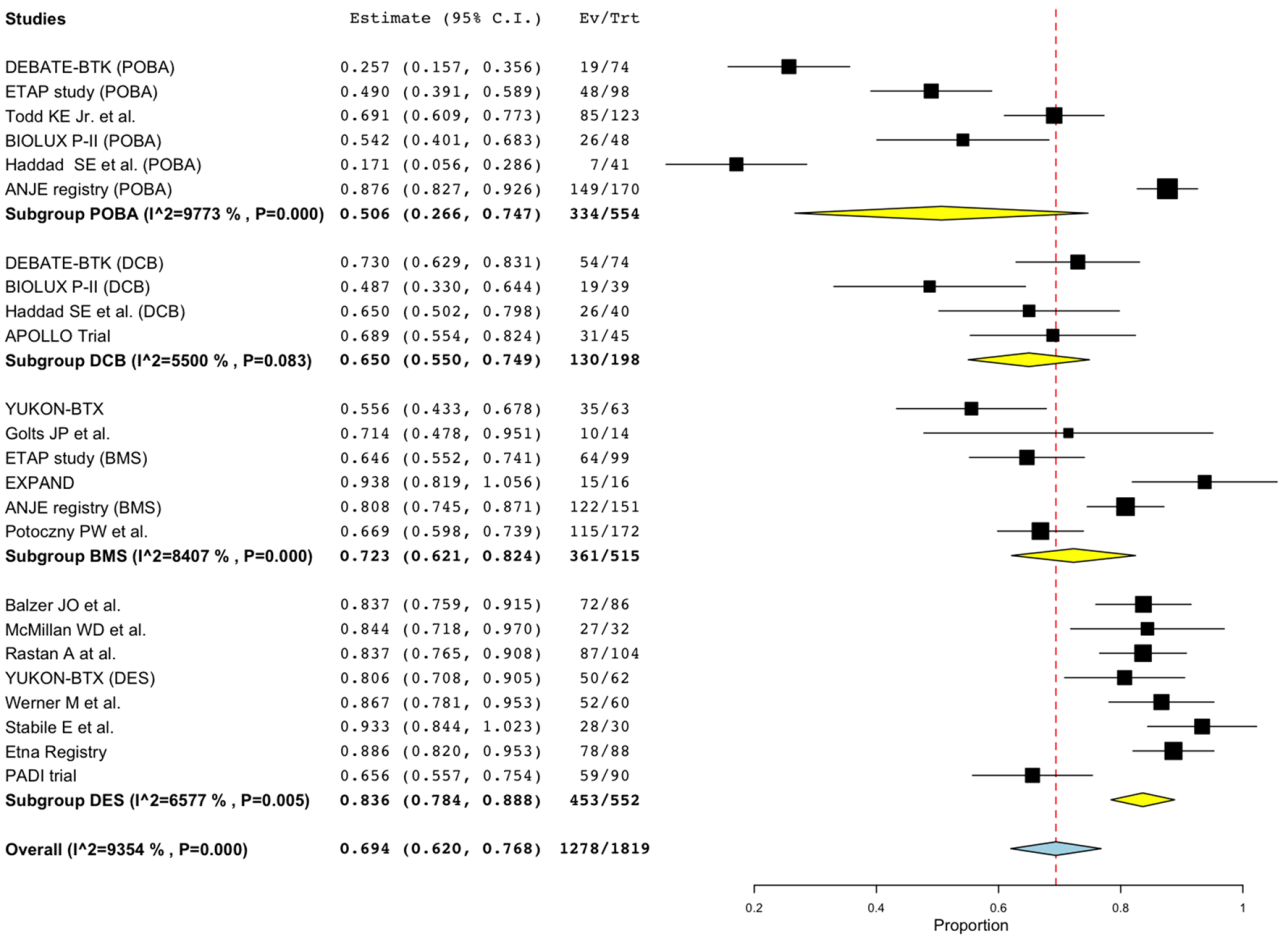
Forest plot of comparisons of 1-year pp by device groups. *POBA* conventional balloon catheters, *DCB* drug-coated balloons, *BMS* bare-metal stents, *DES* drug-eluting stents, *pp* primary patency rates

**Fig. 3:**
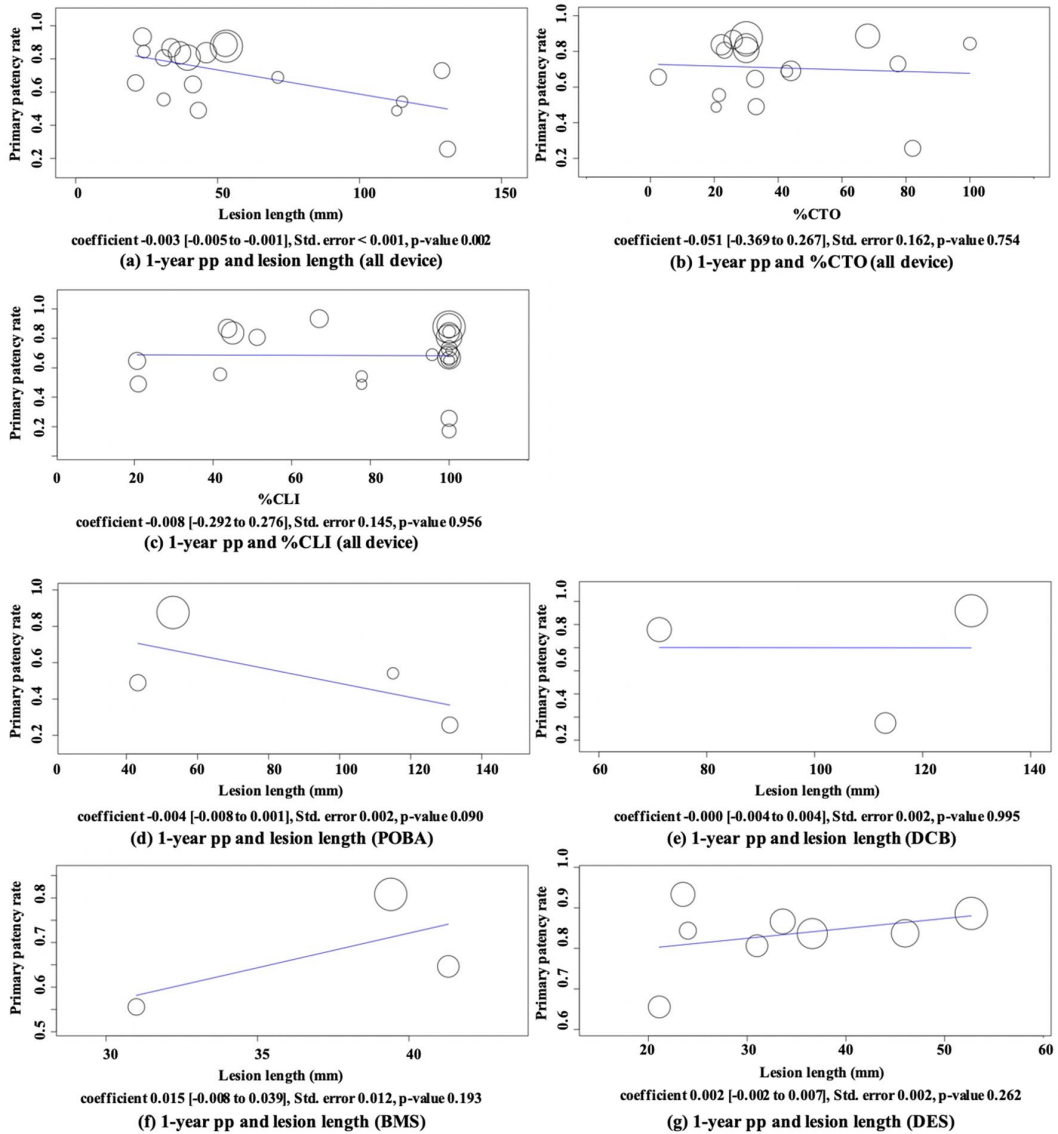
Associations between 1-year pp and lesion characteristics. **a** 1-year pp and lesion length, (**b**) 1-year pp and percentage of CTO lesions, (**c**) 1-year pp and percentage of CLI patients, (**d**) 1-year pp and lesion length with POBA, (**e**) 1-year pp and lesion length with DCB, (**f**) 1-year pp and lesion length with BMS, (**g**) 1-year pp and lesion length with DES. *POBA* conventional balloon catheters, *DCB* drug-coated balloons, *BMS* bare-metal stents, *DES* drug-eluting stents, *pp* primary patency rates, *CTO* chronic total occlusion, *CLI* critical limb ischemia

Pooled estimates for 1-year pp are shown in Fig. 2. Point estimate of 1-year pp of POBA was 50.6% (95% CI 26.6%–74.7%, studies = 6; *I*^2^ = 98%, *P* < 0.001), DCB was 65.0% (95% CI 55.0%–74.9%, studies = 4; *I*^2^ = 55%, *P* = 0.083), BMS was 72.3% (95% CI 62.1%–82.4%, studies = 6; *I*^2^ = 84%, *P* < 0.001), and DES was 83.6% (95% CI 78.4%–88.8%, studies = 8; *I*^2^ = 66%, *P* = 0.005). On excluding the PADI study data, 1-year pp of DES was 86.1% with a narrower confidence interval (95% CI 83.0%–89.2%, studies = 7; *I*^2^ = 0%, *P* = 0.522) [25]. Meta-regression analysis yielded a negative coefficient between the lesion length and 1-year pp, implying that for every 10 mm increase in lesion length, there was a 3% decrease in 1-year pp (*P* = 0.002) (Fig. 3a). There was no statistically significant coefficient between 1-year pp and the ratio of CTO lesions or the ratio of CLI patients in the study population (Fig. 3b, c). The impact of the lesion length was not observed when the analyses were conducted by device groups (Fig. 3d–g). The sample size was small for each device group; therefore, the coefficients might have been impacted by a heavily weighted study.

### Major amputation rate in each device group

**Fig. 4:**
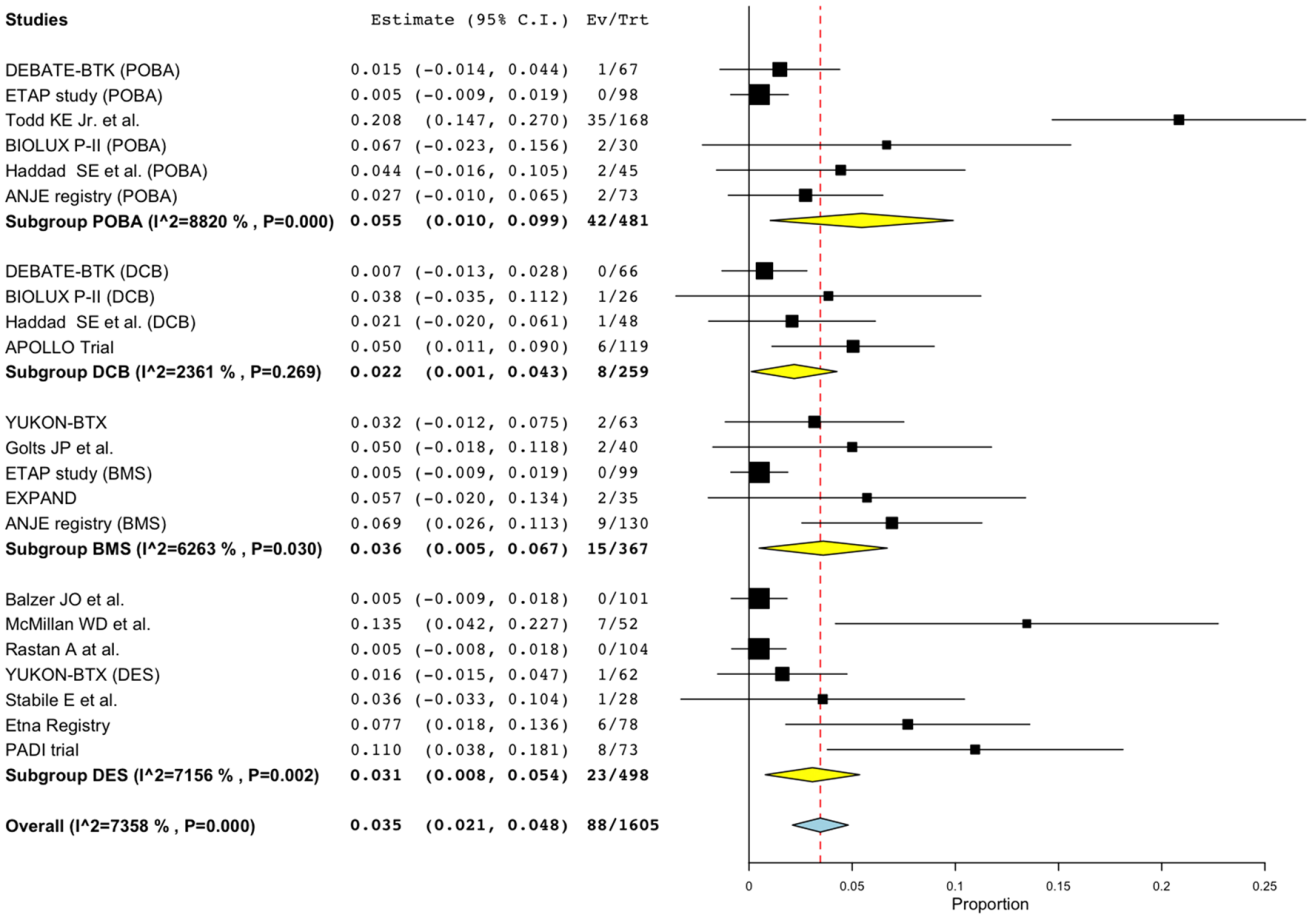
Forest plot of comparisons of 1-year major amputation rates by device groups. *POBA* conventional balloon catheters, *DCB* drug-coated balloons, *BMS* bare-metal stents, *DES* drug-eluting stents

**Fig. 5:**
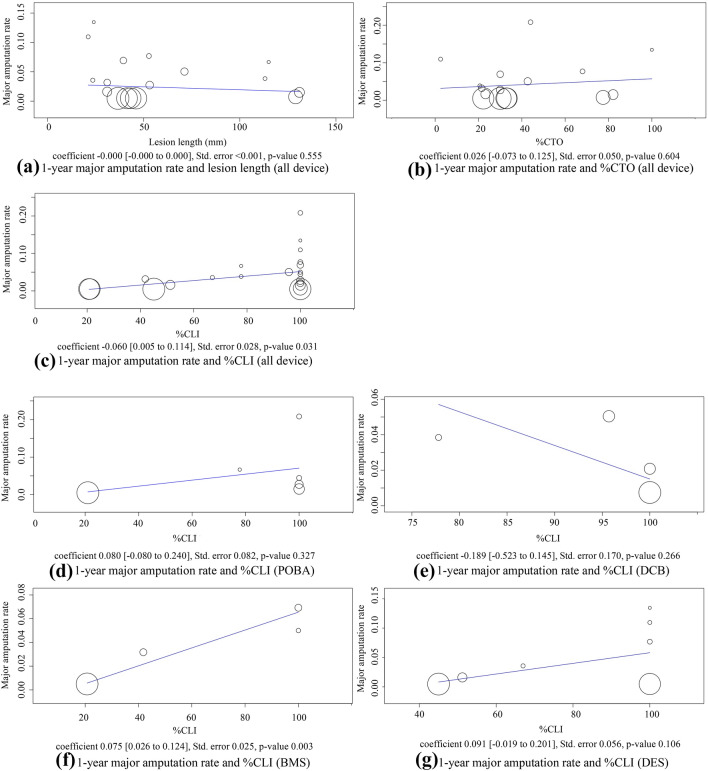
Associations between 1-year major amputation rates and lesion characteristics. **a** 1-year major amputation rates and lesion length, (**b**) 1-year major amputation rates and percentage of CTO lesions, (**c**) 1-year major amputation rates and percentage of CLI patients, (**d**) 1-year major amputation rates and percentage of CLI patients with POBA, (**e**) 1-year major amputation rates and percentage of CLI patients with DCB, (**f**) 1-year major amputation rates and percentage of CLI patients with BMS, (**g**) 1-year major amputation rates and percentage of CLI patients with DES. *POBA* conventional balloon catheters, *DCB* drug-coated balloons, *BMS* bare-metal stents, *DES* drug-eluting stents, *CTO* chronic total occlusion, *CLI* critical limb ischemia

Pooled estimates for 1-year amputation rates are shown in Fig. 4. There was no statistically significant difference between the device groups; point estimate of 1-year amputation rates of POBA was 5.5% (95% CI 1.0%–9.9%, studies = 6; *I*^2^ = 88%, *P* < 0.001), DCB was 2.2% (95% CI 0.1%–4.3%, studies = 4; *I*^2^ = 24%, *P* = 0.269), BMS was 3.6% (95% CI 0.5%–6.7%, studies = 5; *I*^2^ = 63%, *P* = 0.03), and DES was 3.1% (95% CI 0.8%–5.4%, studies = 7; *I*^2^ = 72%, *P* = 0.002). Meta-regression analysis indicated no statistically significant coefficient between 1-year amputation rates and lesion length or the ratio of CTO lesions in the study population (Fig. 5a, b). There was a positive coefficient between the ratio of CLI patients and 1-year amputation rates (*P* = 0.031) (Fig. 5c). This same trend was observed in the sub-group analysis by the device groups except for DCB (Fig. 5d--g). Heavily weighted studies had the lowest amputation rates for each device group and the coefficients might have been impacted by them.

## Discussion

This study sought to evaluate EVT device performance in BTK lesions and the influence of lesion characteristics on the results. Our analysis demonstrated that DES had the best pp among the four test devices (83.6%). There was a negative coefficient between lesion length and pp. The ratio of CLI patients impacted the amputation rates, whereas no statistically significant difference was found between the devices. As DCBs have only been clinically evaluated for about a decade, a study period of 10 years was chosen for this analysis to minimize the bias of technical level of intervention among the devices [4]. To compensate for the limited number of randomized controlled trials (RCTs), non-randomized studies were included in the analysis which resulted in the inclusion of 777 patients in the DES group for this study. Efforts to minimize the risk of bias were made by removing the ISR cases and limiting the rate of provisional stenting in POBA and DCB cases.

In our study, pp were highest with DES, and lesion length affected pp. Considering that only 5 studies had average length of more than 100 mm, clinical study for long lesions is expected to gain a better estimate of the coefficient. On the contrary, there was no statistically significant difference between the devices regarding major amputation rates. The ratio of CLI patients significantly impacted the rates. It is clear that the amputation rate of the DES group was increased by the results from three of the DES cohorts, which showed the highest amputation rates within the DES group and which included CLI patients only [20, 24, 25]. In fact, all three of these studies included a higher rate of Rutherford class 5 and 6 patients: 82% in McMillan’s study, 61% in Etna Registry, and 87% in PADI study. Even though Rutherford classes 4 to 6 are all categorized as CLI, there is a distinct difference in clinical presentations between Rutherford class 4 and classes 5–6. Patients diagnosed with Rutherford classes 5–6 have some form of tissue damage/loss in their foot and/or leg, whereas clinical presentation of Rutherford class 4 is rest pain without tissue loss. The degree of tissue loss correlates with the likelihood of a longer healing time as well as amputation [28]. Patients with Rutherford class 4 require significantly less management and are less likely to lose a limb in the short term. The Society for Vascular Surgery (SVS) documented that the risk stratification based on wound, ischemia, and foot infection (WIfI) for CLI patients. In the SVS WIfI scoring system for the likelihood of amputation, patients with rest pain are considered at low or very low risk of limb loss [28]. Therefore, the high rate of Rutherford classes 5–6 patients in the three studies presumably affected the DES amputation rate. The other study with only CLI patients in the DES group was Balzer’s study; however, only 46% were Rutherford class 5 and the rest were Rutherford class 4. This suggests that stricter patient background matching was required to assess the risk of amputation rates by meta-analysis because not only the Rutherford class but multiple factors, such as renal insufficiency, malnutrition, untreated infection, and control of diabetes, can affect the degree of ulcer and thus, the requirement of amputation [28].

In addition to average lesion length, the ratio of CTO lesions, and the ratio of CLI patients, an effort was made to investigate the impact of the ratio of calcified lesions and medications on the outcomes. However, an analysis of any possible correlation between the percentages of calcified lesion and pp or amputation rates could not be conducted because of limited availability of data. Except for one POBA cohort and one BMS cohort, information about dual anti-platelet therapy (DAPT) after the interventions were obtained from all other cohorts (Table 1). In general, shorter DAPT durations are preferable for patients to reduce the risk of bleeding. A minimum of 2 months-DAPT is currently recommended in the instructions for use of DES for above-the-knee lesions. However, longer DAPT is usually prescribed for patients with DES because of a possible delayed healing process caused by anti-proliferative drugs applied on the stents. In this study, 6-month DAPT was prescribed for 7 out of 8 cohorts in DES group, and 3 months in 1 cohort (Etna registry). The 3-month cohort showed similar favorable pp as the 6-month cohorts suggesting that longer DAPT durations may not be required for DES. No correlation was found between the duration of DAPT and amputation rates.

All of the stents in the included studies were coronary or superficial femoral artery stents, which limits the target lesion length of BMS and DES groups. Combined with the fact that the lesion lengths treated with stents (BMS, DES) were shorter than those with balloons (POBA, DCB), and the lesion length was negatively affected at 1-year pp, the superiority of DES over DCB might be biased to some extent. However, meta-regression analysis in the DES group did not display a negative coefficient between 1-year pp and lesion length, suggesting its potential performance benefits in long lesions. The 1-year pp values from the PADI study were much lower than those from other DES studies [25]. As mentioned previously, the population of the PADI study included the highest percentage of Rutherford classes 5–6 patients among the DES cohort. These data indicated that the vessel condition of the target lesion of the patients in the PADI study was worse than those in other studies. Moreover, the PSVR cut-off value for being patent or restenosed was 2.0 which is lower than in other studies. Those conditions might have led to the low pp in the PADI study [25]. It is important to address the fact that pp of McMillan’s study and Etna Registry were aligned with those of other DES cohorts even though they showed high rates of amputation [20, 24]. As described previously, those studies included high rates of Rutherford class 5 and 6 patients, the same as the PADI study. As defined in WIfI, tissue-related factors, such as wound management and infection management, as well as the degree of ischemia determine the risk of amputation. The degree of tissue loss might have affected the higher rates of amputation. Freedom from amputation rate is not correlated with patency, because time to wound healing varies depending on the depth and area of wound or infections [28]. However, quickly restoring blood circulation to the affected part of the foot is certainly important to cure gangrene and it should be a significant factor in promoting higher limb salvage rates.

DES developed exclusively for BTK lesions are still not available. Several meta-analyses have demonstrated practical performance of coronary DES in BTK lesions. Biondi-Zoccai et al. performed a meta-analysis including 640 patients (*n* = 272 for DES arm) from 18 non-randomized studies on stents (BMS and DES) for CLI patients in 2009 [1]; they concluded that sirolimus-eluting stents were superior to BMS in primary patency after a median follow-up of 12 months. However, there was no statistically significant difference in limb salvage between DES and BMS. One of the limitations of the meta-analysis was that the mean follow-up for 7 of the 18 studies was less than 12 months. Regarding meta-analysis of RCTs, Fusaro et al. analyzed 611 patients from 5 RCTs on comparison of DES (*n* = 294) to control devices (POBA or BMS) in 2013 [2]. Six years later, Varcoe et al. conducted a meta-analysis of 7 RCTs that enrolled 810 patients comparing DES (*n* = 387) and control devices, which added an additional 2 RCTs to the meta-analysis by Fusaro et al. [3]. At 12 months, DES gave better results for target lesion revascularization, patency, and amputation in both meta-analyses, although improvement of Rutherford class for DES was statistically better only in the analysis by Varcoe et al. No difference in mortality rates was found between DES and the control devices in either meta-analysis. In 2016, Katsanos et al. reported on a Bayesian network meta-analysis of 16 RCTs comprising 1805 patients comparing DES (5 RCTs, *n* = 394), BMS, and POBA [4]. Restenosis, target lesion revascularization, amputation, and wound healing were favorable for DES in their study. All three meta-analyses indicated the efficacy of DES in BTK lesions; however, the effectiveness of DES on each endpoint varied to some extent. Clinical data for BTK lesions is still limited, and long-term follow-up results from large RCTs are necessary to better understand the relevance of EVT to clinical outcomes.

In general, maintaining the patency in BTK lesions after intervention is more difficult compared with above-the-knee lesions due to its lesion characteristics, such as smaller diameters, slower blood flow, and presence of calcium-rich lesions in the tunica media [29, 30]. Considering that lesion length of BTK lesions tends to be long [30], pp in long lesions requires improvement. Since coronary DES have shown consistently good patency in short BTK lesions, it could also be possible to achieve positive results in longer lesions with DES. Conversely, stent implantation in BTK lesions is still debatable as it increases the risk of thrombus formation and restenosis. Use of a novel long DES with exceptional anti-thrombogenicity or use of a combination of DES and atherectomy devices might improve pp in long BTK lesions. Optimal EVT for long BTK lesions still needs to be explored.
